# Identification and Characterization of Proteins That Are Involved in RTP1S-Dependent Transport of Olfactory Receptors

**DOI:** 10.3390/ijms24097829

**Published:** 2023-04-25

**Authors:** Ryosuke Inoue, Yosuke Fukutani, Tatsuya Niwa, Hiroaki Matsunami, Masafumi Yohda

**Affiliations:** 1 Department of Biotechnology and Life Science, Tokyo University of Agriculture and Technology, Koganei, Tokyo 184-8588, Japan; ryosuke.inoue@yohda.net (R.I.); fukutani@cc.tuat.ac.jp (Y.F.); 2 Cell Biology Center, Institute of Innovative Research, Tokyo Institute of Technology, Yokohama 226-8503, Japan; tniwa@bio.titech.ac.jp; 3 Department of Molecular Genetics and Microbiology, Duke University Medical Center, Durham, NC 27710, USA; hiroaki.matsunami@duke.edu

**Keywords:** odorant receptor, receptor-transporting protein, membrane traffic, proximity biotinylation, chaperone

## Abstract

Olfaction is mediated via olfactory receptors (ORs) that are expressed on the cilia membrane of olfactory sensory neurons in the olfactory epithelium. The functional expression of most ORs requires the assistance of receptor-transporting proteins (RTPs). We examined the interactome of RTP1S and OR via proximity biotinylation. Deubiquitinating protein VCIP135, the F-actin-capping protein sub-unit alpha-2, and insulin-like growth factor 2 mRNA-binding protein 2 were biotinylated via AirID fused with OR, RTP1S-AirID biotinylated heat shock protein A6 (HSPA6), and double-stranded RNA-binding protein Staufen homolog 2 (STAU2). Co-expression of HSPA6 partially enhanced the surface expression of Olfr544. The surface expression of Olfr544 increased by 50–80%. This effect was also observed when RTP1S was co-expressed. Almost identical results were obtained from the co-expression of STAU2. The interactions of HSPA6 and STAU2 with RTP1S were examined using a NanoBit assay. The results show that the RTP1S N-terminus interacted with the C-terminal domain of HSP6A and the N-terminal domain of STAU2. In contrast, OR did not significantly interact with STAU2 and HSPA6. Thus, HSP6A and STAU2 appear to be involved in the process of OR traffic through interaction with RTP1S.

## 1. Introduction

Olfaction is mediated via olfactory receptors (ORs) that are situated on the cilia membrane of olfactory sensory neurons (OSNs) in the olfactory epithelium [1,2]. ORs detect chemicals in the air and transmit the information directly to glomeruli in the olfactory bulb, after which they relay the information to higher brain regions where the perception of odor is constructed [3]. ORs are a type of G-protein-coupled receptor (GPCR) and constitute the largest family of these receptors. Humans have approximately 400 functional OR genes, whereas mice have over 1100 [4,5]. Each mature OSN expresses only one OR in accordance with the “one cell–one receptor” rule [6], which is critical for OSN maturation [7]. However, many ORs fail to be transported into the plasma membrane and instead remain in the endoplasmic reticulum when expressed in non-olfactory cells [8,9]. To address this issue, receptor-transporting proteins (RTPs), such as RTP1S (the short form of RTP1) and RTP2, act as chaperones and mediate the transport of ORs to the plasma membrane of OSNs. RTPs play essential roles in OR expression and OR gene choice [10,11,12]. RTPs, when expressed in mammalian cells or yeasts, aid in the transport and functional expression of various ORs to the plasma membrane [13]. This discovery enabled the functional characterization of ORs, including high-throughput ligand screening [14,15,16,17,18]. RTP1S and RTP2 are abundantly expressed in olfactory tissues and may also exist in other tissues where functional ORs are ectopically present. RTP3 and RTP4, on the other hand, are expressed in non-olfactory organs and play a role in expressing other GPCRs [19,20]. No RTP homologs have been found outside of vertebrates [10]. In addition to RTPs, receptor expression-enhancing protein 1 (REEP 1) also serves as an accessory molecule to enhance the expression of olfactory receptors [10]. Hsc70t, a testis-enriched variant of the Hsp70 family of heat shock proteins, was shown to enhance OR expression [21]. However, its effects are relatively limited compared with RTP1S.

The RTP family proteins are type II transmembrane proteins with a cytoplasmic N-terminus and a transmembrane domain (TM domain) close to the C-terminus. The TM domain and the outer cellular region of RTP1S appear to be the signal for promoting OR localization in a lipid raft on the cell membrane [22]. Although the TM domain was thought to be essential for the complete function of RTP1S, TM domain deletion mutants (RTP1S_C3 (1–198 amino acids) and RTP1S_C2 (1–176 amino acids)) partially retained the ability to promote OR localization in the plasma membrane [22]. Immunoprecipitation analysis indicated that RTP1S formed complexes with ORs [22]. The recombinant intracellular domain of RTP1S formed a dimer; there is evidence for RTP1S dimer formation in mammalian cells [23]. However, it is unknown whether RTP1S functions as a dimer.

In RTP1 and RTP2 double-knockout mice, the majority of ORs are significantly under-represented (uORs) and the mature OSNs expressing them are lost, suggesting that these ORs require RTP1 and RTP2 for functional expression [12]. Interestingly, a small sub-set of ORs is over-represented (oORs), meaning that a minor sub-set of ORs function without RTP1 and RTP2. Accordingly, some oORs show cell surface expression when expressed without RTPs in heterologous cells [12]. The instability of OR proteins is likely to cause poor cell surface expression of ORs [24]. It is reasonable to think that RTP1 and RTP2 interact and stabilize ORs to enhance their surface expression. However, unlike canonical chaperons RTPs lack similarities with other known chaperones. One possible way to explain the RTP mode of action is via the recruitment of canonical chaperones, which assist in OR folding. To test this hypothesis, we took an unbiased approach to identify potential interacting proteins using the BioID proximity labeling method. We found that a molecular chaperone—heat shock 70 kDa protein 6 (HSPA6)—and double-stranded RNA-binding protein—Staufen homolog 2 (STAU2)—are biotinylated through biotin ligase fused with RTP1S in the presence of OR. Thus, the function of STAU2 and HSPA6 in OR expression is elucidated herein.

## 2. Results

### 2.1. Design and Construction of RTP1S and OR Fused with Biotin Ligase

**Figure 1:**
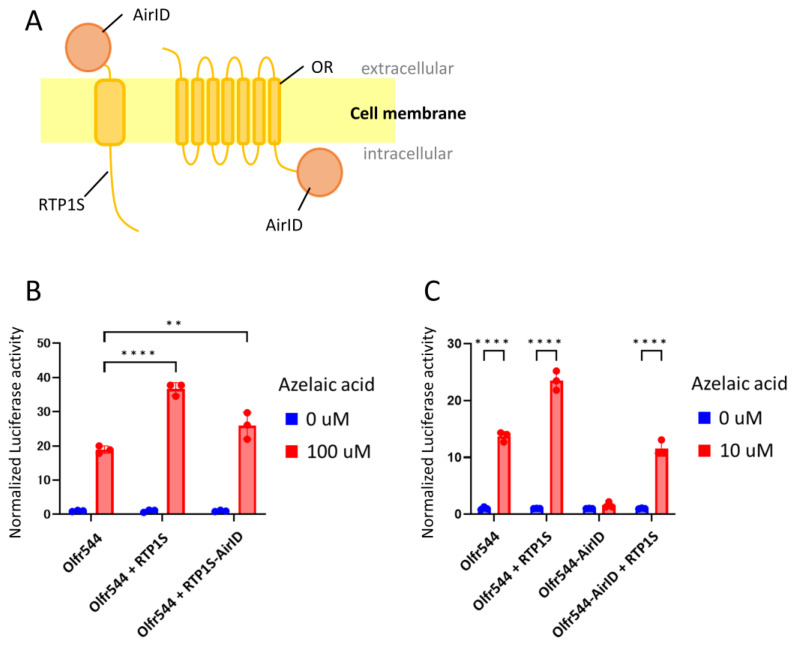
Effect of AirID fusion on RTP1S and Olfr544. (**A**) Schematic drawings of AirID-fused RTP1S and OR. (**B**) Ligand responses of Olfr544 with RTP1S and RTP1S-AirID. (**C**) Ligand response of Olfr544 and Olfr544-AirID with or without RTP1S. The ligand response was analyzed with a Dual-Glo^®^ luciferase assay using azelaic acid as a ligand. The luciferase activities of each condition were calculated as fold change between the response at 0 μM and 100 μM (**A**) or 10 μM (**B**). Each comparison was performed in triplicate. Multiple comparisons were performed using two-way analysis of variance (ANOVA) followed by Tukey test (** *p* < 0.01, **** *p* < 0.0001).

We used AirID biotin ligase for BioID. AirID is a biotin ligase designed via the ancestral enzyme reconstruction algorithm [25]. Although its biotinylation activity is lower than that of other biotin ligases, non-specific biotinylation is reduced. AirID was fused to the C-termini of RTP1S and a mouse OR—Olfr544—because modification of the N-termini of RTP1S seriously affected its function in our preliminary experiments. The C-terminus of RTP1S was exposed to the extracellular region across the cell membrane (Figure 1A). In contrast, the C-terminus of OR is located in the intracellular region (Figure 1A). To examine the functionality of AirID-fused RTP1S (RTP1S-AirID) and AirID-fused Olfr544 (Olfr544-AirID), we expressed them in HEK293T cells and measured the ligand response of Olfr544 with a Dual-Glo luciferase assay using azelaic acid as a ligand (Figure 1B). Although the effect of RTP1S was significantly affected by the fusion, the ligand response of Olfr544 was observed in the presence of RTP1S-AirID. Olfr544-AirID exhibited a signal response when RTP1S was co-expressed (Figure 1C).

### 2.2. Proximity Labeling-Coupled Mass Spectrometry in HEK293T Cells

To screen the proteins that interact with RTP1S and OR, we performed proximity labeling in HEK293T cells. HEK293T cells were transfected with the four sets of clones of Olfr544-AirID, RTP1S-AirID, Olfr544-AirID + RTP1S, and Olfr544 + RTP1S-AirID and then incubated with biotin for 24 h. The biotinylated proteins in the cell lysate were collected using Streptavidin Mag Sepharose and then applied for LC-MS/MS after trypsin digestion. The identified proteins are shown in Supplementary Dataset S1.

**Figure 2:**
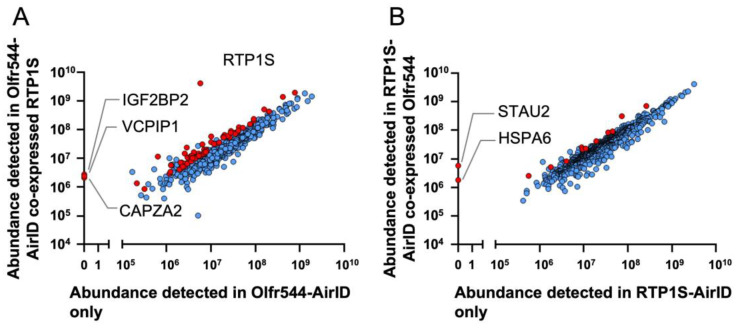
Scatter plots of relative abundance of proteins detected in BioID. Each protein was plotted using number of peptides detected via BioID. Red symbol represents protein whose abundance ratio was ≥2 and adjusted *p*-value was <0.05. (**A**) Comparison between Olfr544-AirID only and Olfr544-AirID with RTP1S. (**B**) Comparison between RTP1S-AirID only and RTP1S-AirID with Olfr544.

We then examined the effect of the co-expression of Olfr544 and RTP1S. Among the proteins biotinylated under the Olfr544-AirID-only condition, 67 proteins, including RTP1S, showed statistically increased detection when co-expressed with RTP1S compared to only Olfr544-AirID (Abundance Ratio ≥ 2 and adjusted *p*-value < 0.05) (Figure 2A). Among them, deubiquitinating protein VCIP135 (VCPIP135), F-actin-capping protein sub-unit alpha-2 (CAPZA2), and insulin-like growth factor 2 mRNA-binding protein 2 (IGF2BP2) were detected only when RTP1S-AirID and Olfr544 were co-expressed. Co-expression of Olfr544 increased the detection of 13 proteins biotinylated via RTP1S-AirID (Figure 2B). Heat shock protein A6 (HSPA6) and double-stranded RNA-binding protein Staufen homolog 2 (STAU2) were specifically detected when RTP1S-AirID was co-expressed with Olfr544.

Among the five proteins specifically detected in the co-expression of Olfr544 and RTP1S, we decided to focus on HSPA6, which is a canonical molecular chaperone, and STAU2, which was detected under the same conditions. HSPA6, also known as Hsp70-6 or Hsp70B, is a molecular chaperone that belongs to the Hsp70 family. HSP70s are a family of conserved ubiquitously expressed heat shock proteins that play an essential role in protein quality control by ensuring that clients fold correctly. Some Hsp70s, such as Hsp70-1a, are expressed against various stresses, including heat and oxidative stresses. In contrast, others, such as Hsc70, are constitutively expressed and involved in proteins’ transport degradation and folding [26]. Hsc70t is a testis-enriched variant of the Hsp70 family of heat shock proteins. Hsc70t also expresses in post-meiotic germ cells in the testis [21]. Co-expression of Hsc70t significantly enhances OR expression in HEK293 cells. Staufen is an RNA-binding protein that is important for mRNA localization and translation in various organisms. Staufen was identified as an essential factor for the proper localization of mRNAs in Drosophila oocyte, such as osker mRNA and bicoid mRNA [27,28,29]. Two homologs are identified in mammals: the ubiquitously expressed STAU1 and the brain-abundant STAU2 [30]; Staufen was shown to be involved in mRNA transport and localization in neuronal cells [31].

### 2.3. HSPA6 and STAU2 Facilitate Surface Expression and Ligand Response of Olfr544

**Figure 3:**
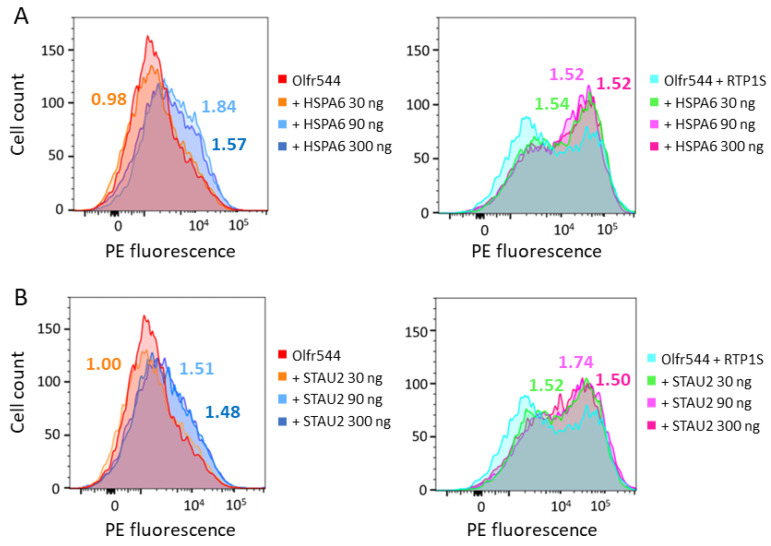
Effects of co-expression of HSPA6 or STAU on cell surface expression of Olfr544. FACS analysis for cell surface expression of Rho-Olfr544 co-expressed with (**A**) HSPA6 (left), HSPA6 and RTP1S (right), (**B**) STAU2 (left), STAU2, and RTP1S (right). Numbers on the graphs represent geometric means relative to examples without HSPA6 or STAU2.

We examined the effect of HSPA6 and STAU2 on the surface expression of ORs. The expression level of ORs on the plasma membrane with the co-expression of HSPA6 or STAU2 was measured using FACS. The dose of HSPA6 and STAU2 was altered from 1/3 to 1/30 of the ORs (30–300 ng/well). Co-expression of HSPA6 partially enhanced the surface expression of Olfr544 (Figure 3A Left). With HSPA6, the geometric mean of PE-fluorescence, which represents the degree of surface expression of Olfr544, was increased by 50–80% compared to without HSPA6. A similar effect was observed when RTP1S was co-expressed (Figure 3A Right). Almost identical results were obtained via the co-expression of STAU2 (Figure 3B).

**Figure 4:**
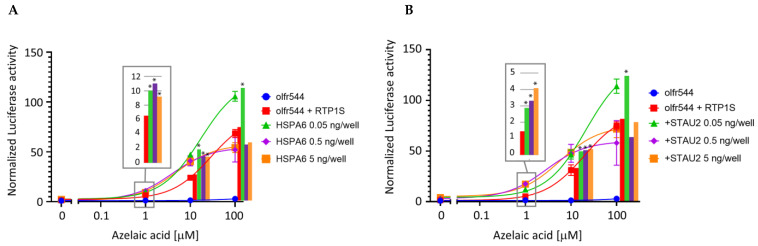
Effects of co-expression of HSPA6 or STAU2 on ligand response of Olfr544. Ligand responses of Olfr544 co-transfected with RTP1S and HSPA6 (**A**) or STAU2 (**B**) were examined with a Dual-Glo luciferase assay and normalized with that of co-expression of Olfr544 and RTP1S only. Dunnett’s test was performed to test whether each luciferase intensity in co-expression with HSPA6 or STAU2 exceeded that observed in co-expression with Olfr544 and RTP1S only (* *p* < 0.05). Each comparison was performed in triplicate.

Interestingly, a relatively high increase in Olfr544 membrane expression was observed in the low-dose condition of HSPA6 or STAU2 when RTP1S was co-expressed. On the other hand, HSPA6 and STAU2 had almost no effect on the surface expression of the other ORs, i.e., Olfr599 and Olfr1484 (Supplementary Figure S1). This difference may be due to the RTP dependency. Although RTP1S enhanced the surface expression of Olfr544, partial expression was observed without RTP1S co-expression. On the contrary, Olfr599 and Olfr1484 were stringently dependent on RTPs. Since HSPA6 and STAU2 were highly biotinylated in the presence of both OR and RTP1S, they appear to be mainly involved in RTP1S-assisted OR expression. Using the Dual-Glo Luciferase assay, we also measured the effect of HSPA6 or STAU2 on the ligand response of Olfr544 in the presence of RTP1S (Figure 4). The dose of STAU2 or HSPA6 was set to 1/1 (5 ng/well), 1/10 (500 pg/well), or 1/100 (50 pg/well) of that of Olfr544. Both HSPA6 and STAU2 significantly enhanced the response of Olfr544 to azelaic acid at the lowest dose, i.e., 50 pg/well.

### 2.4. Intracellular Interaction Analysis Using NanoBiT Assay

We examined interactions among OR, RTP1S, HSPA6, and STAU2 with the binary split luciferase complementation method using NanoBiT^®^: NanoLuc^®^ Binary Technology [32]. In this method, split NanoLuc Luciferase fragments, as well as a larger protein domain (Lg) and a small peptide (Sm), were fused with proteins; this interaction was the subject of study. We constructed genes for Olfr544, RTP1S, HSPA6, and STAU2 connected with Lg or Sm at the N- or C-termini and expressed them in HEK293T cells using a pCI vector. They were named as follows: Lg fusions at the N-terminus (Lg-544, Lg-RTP1S, Lg-HSPA6, Lg-STAU2); Sm fusions at the N-terminus (Sm-544, Sm-RTP1S, Sm-HSPA6, Sm-STAU2); Lg fusions at the C-terminus (544-Lg, RTP1S-Lg, HSPA6-Lg, STAU2-Lg); and Sm fusions at the C-terminus (544-Sm, RTP1S-Sm, HSPA6-Sm, STAU2-Sm) (Supplementary Figure S2).

**Figure 5:**
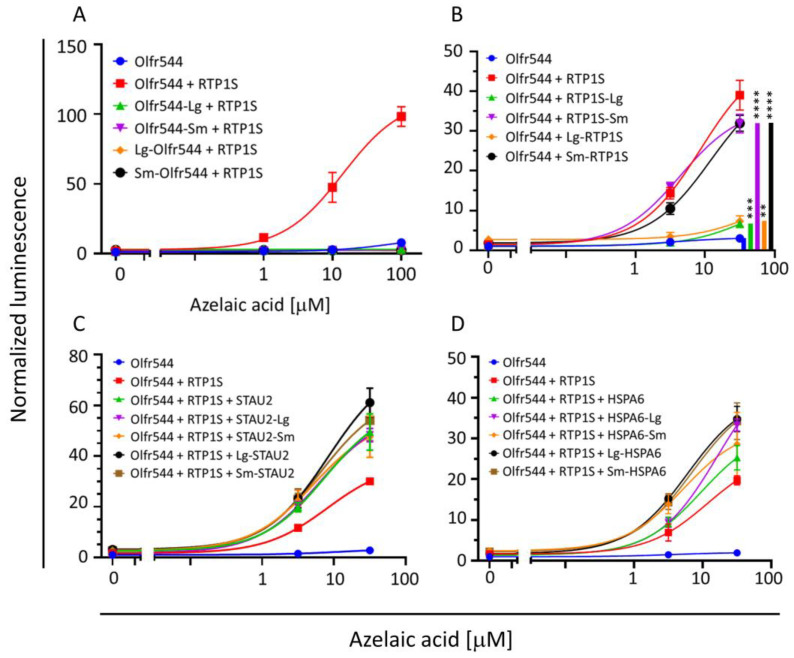
Effects of NanoBiT fusion with a NanoBiT sub-unit on Olfr544, RTP1S, and HSPA6. Dual-Glo luciferase assay to detect effects of the addition of NanoBiT sub-unit on functions of each protein. (**A**) Olfr544; (**B**) RTP1S. Statistical comparisons were performed using Student t-test against Olfr544. ** *p* < 0.01, *** *p* < 0.001, **** *p* < 0.0001). (**C**) STAU2; (**D**) HSPA6. Each comparison was performed in triplicate.

We examined the functionality of the fused proteins via the ligand responses of Olfr544. The fusion significantly affected the ligand responses of all Olfr544 variants (Figure 5A). Olfr544 connected with Sm and Lg at the C-termini exhibited a slight ligand response; however, the N-terminal fusion mutants of Olf544 did not exhibit a similar response (Supplementary Figure S3). The connection of Sm to RTP1S on both termini did not affect the ability to assist the functional expression of Olfr544. However, the fusion with Lg significantly affected the chaperone activity of RTP1S (Figure 5B). The abilities of STAU2 and HSPA6 were not significantly affected by the addition of split fragments on both termini (Figure 5C,D).

**Figure 6:**
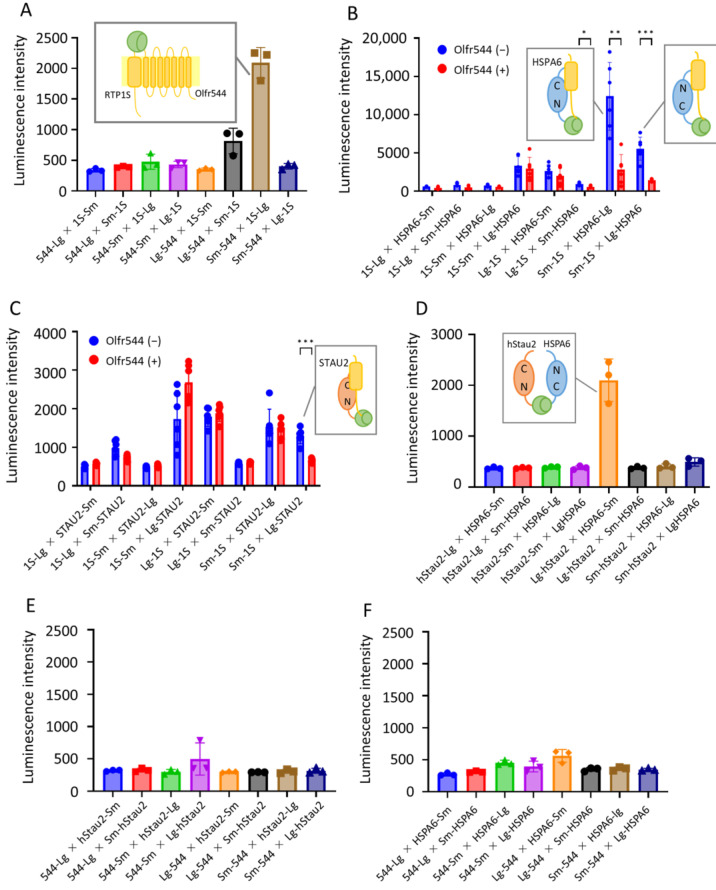
NanoBit assay for interactions among OR, RTP1S, HSPA6, and STAU2. Luminescence intensities were measured with a NanoBit assay using various combinations of fusion proteins. (**A**) Between Olfr544 and RTP1S; (**B**) between RTP1S and HSPA6 with or without Olfr544; (**C**) between RTP1S and STAU2 with or without Olfr544; (**D**) between HSPA6 and STAU2; (**E**) Olfr544 and HSPA6; and (**F**) Olfr544 and STAU2. Error bar indicates S.E.M. (n 3 to 6). N = 3. In (**B**) and (**C**), statistical comparisons between Olfr544(−) and Olfr544(+) were performed using multiple t-test with Holm–Šídák method (* *p* < 0.05, ** *p* < 0.01, *** *p* < 0.001). N = 6.

We examined the interaction between Olfr544 and RTP1S using the NanoBit assay. Among the eight combinations, Olfr544 with N-terminal fusion and RTP1S with C-terminal fusion exhibited an increase in luminescence (Figure 6A). In particular, significant luminescence was observed in Sm-544 and RTP1S-Lg. Thus, the N-terminus of Olfr544 must interact with the C-terminus of RTP1S, which is reasonable since both the N-terminus of OR and the C-terminus of RTP1S are exposed outside of the plasma membrane. Next, we performed NanoBit assays on RTP1S and HSPA6 with and without Olfr544 (Figure 6B). In the absence of Olfr544, notable interactions between the RTP1S N-terminus and HSP6A were observed. The combination of Sm-RTP1S and HSPA6-Lg showed higher luminescence. Interestingly, luminescence was almost suppressed in the presence of Olfr544. Olfr544 did not interfere with the interaction between RTP1S and STAU2, with the exception of the interaction between Sm-RTP1S and Lg-STAU2 (Figure 6C). Therefore, the N-terminal region of RTP1S, which is located on the cytosol side of the membrane, interacted with HSPA6 and the N-terminal domain of STAU2. Consistently, the combination of Lg-STAU2 and HSPA6-Sm produced significant luminescence, suggesting a complex formation (Figure 6D). The OR did not exhibit a significant interaction with STAU2 and HSPA6 (Figure 6E,F). The interaction of OR and RTP1S supports their complex formation, inhibiting the interaction of RTP1S with HSPA6 and STAU2. These results contradict the results of the BioID experiments in which the interaction of RTP1S with HSPA6 and STAU2 was observed only when OR was co-expressed. In the BioID experiments, biotin ligase was fused at the C-terminus of RTP1S. As the C-terminus is located on the other side of the N-terminus, it is consistent that STAU2 and HSPA6 were not observed in the BioID experiments without OR. The fact that STAU2 and HSPA6 were only biotinylated in the presence of OR suggests that the N-terminus of RTP1S comes across with HSPA6 and STAU2 during the trafficking process of OR.

## 3. Discussion

Five proteins were selectively biotinylated in the proximity labeling experiments when OR and RTP1S were co-expressed. Deubiquitinating protein VCIP135, the F-actin-capping protein sub-unit alpha-2, and insulin-like growth factor 2 mRNA-binding protein 2 were biotinylated via AirID fused with OR, RTP1S-AirID biotinylated heat shock protein A6 (HSPA6), and double-stranded RNA-binding protein Staufen homolog 2 (STAU2). At first, we considered the function of HSPA6 since Hsp70 functions as a chaperone for various proteins. BiP is an Hsp70 in the endoplasmic reticulum, where secreted proteins and membrane-bound proteins fold. Hsc70t, a testis-enriched variant of the Hsp70 family of heat shock proteins, enhanced OR expression. There is a possibility that HSPBA6 recruited to RTP1S to stabilize the ORs. We then examined the effect of the co-expression of an Hsp70, HspA1L. Similar to HSPA6, HspA1L itself did not enhance OR expression. Although HSPA6 enhanced the surface expression of OR in the presence of RTP1S, the expression of HspA1L partially suppressed OR expression. This result suggests that HspA1L interferes with the transport of OR via the interaction with RTP1S or OR.

Staufen is a double-stranded RNA-binding protein first described in Drosophila oocytes as an essential regulator of the posterior–anterior localization of mRNAs. It is difficult to conclude that Staufen assists in transporting ORs. Interestingly, there is a report that the localization of translationally silenced Beta2-AR mRNA to the peripheral cytoplasmic regions is critical for receptor localization to the plasma membrane [33]. Beta2-AR mRNA is recognized through the nucleocytoplasmic shuttling RNA-binding protein HuR. In addition, a proteome analysis revealed that Staufen is expressed in mouse olfactory cilia albeit in trace amounts [33]. Thus, Staufen might transport OR mRNA for the membrane localization of ORs [34]. There are two independent Staufen genes in vertebrates, including mammals, fish, amphibians, and birds [35]. Staufen1 (STAU1) and Staufen2 (STAU2) play distinct roles in cellular functions. Despite some similarities in their sequences and RNA-binding domains, only ~30% overlap has been observed in the mRNA contents of their messenger ribonucleoprotein (mRNP) complexes. Moreover, STAU1 is ubiquitously expressed in most cell types and tissues, while STAU2 is predominantly expressed in the brain and heart. The NanoBit experiment clearly showed that the N-terminus of OR and the C-terminus of RTP1S interact; both of these factors are thought to be outside of the plasma membrane. The HSPA6 interacted with the N-terminus of RTP1S. As HSPA6 is located in the cytoplasmic space, the interaction should occur in the cytoplasm before RTP1S integration into the membrane. A relatively strong interaction was observed between the C-terminus of HSPA6 and the N-terminus of RTP1S, likely because the substrate-binding domain is located in the C-terminus of HSPA6. The co-expression of OR suppresses the interaction; thus, it is reasonable to think that HSPA6 interacts with RTP1S before interacting with OR. However, the interaction of STAU2 and RTP1S seems to be more complex. The N-terminus of STAU2 showed interaction with RTP1S, while the C-terminus of RTP1S interacted strongly with the C-terminus of STAU2 when OR was co-expressed. In contrast, the interaction between the N-terminus of RTP1S and the N-terminus of STAU2 decreased with OR co-expression.

The mechanism through which GPCRs are exported from intracellular stores to the plasma membrane is still unknown. It requires a complex network of interactions between GPCRs and either chaperones or escort proteins. The RTPs are regarded as the escort proteins for ORs and taste receptors. The fact that RTP1S dependency correlates with structural stability but no specific interaction motif indicates the chaperone-like function of RTP1S. However, there are no data to show the chaperone-like activity of RTP1S. This study suggests the involvement of other proteins, including a canonical chaperone known as HSPA6. Although other candidates are not regarded as molecular chaperones, their chaperone-like activities should be investigated. It is necessary to examine their interactions with ORs in detail to understand RTP1S-dependent traffic to the membrane.

## 4. Materials and Methods

### 4.1. DNA and Vector Preparation

RTP1S and ORs were expressed using a pCI-vector. ORFs of hStau1, hStau2, HSPA6, and HspA1L were amplified via PCR and sub-cloned into the pCI vector. DNAs for all fusion proteins were constructed via in-fusion PCR. These DNAs were sub-cloned into the pCI expression vector. All plasmid sequences were verified using Sanger sequencing.

### 4.2. Cell Culture

HEK293T Cell Line used in this study was obtained from Thermo Fisher Scientific (Waltham, MA, USA) (Cat# HCL4517). HEK293T cells were maintained in minimum essential medium Eagle (FUJIFILM Wako Pure Chemical, Osaka, Japan) containing 10% fetal bovine serum (Biowest, Nuaillé, France) and 0.5% Antibiotic–Antimycotic Mixed Stock Solution (Nakalai tesque, Kyoto, Japan) at 37 °C with 5% CO_2_.

### 4.3. Luciferase Assay

The Dual-Glo^®^ luciferase assay (Promega, Madison, WI, USA) was used to measure the ligand response of OR. Firefly luciferase, driven by a cAMP response element promoter (CRE-Luc; Stratagene, San Diego, CA, USA), was used to measure the OR activation levels. Renilla luciferase, driven by a constitutively active SV40 promoter (pRL-SV40), was used as an internal control for cell viability and transfection efficiency. HEK293T cells were plated on poly-D-lysine-coated 96-well plates (Thermo Fisher Scientific, Waltham, MA, USA). Plasmid DNAs of each protein and two luciferase constructs were transfected. At 24 h post-transfection, the medium was replaced with 25 µL of odorant solution diluted in CD293 (Thermo Fisher Scientific) and incubated for 3 h at 37 °C. We followed the manufacturer’s protocols for measuring firefly luciferase (Luc) and Renilla luciferase (RL) activities.

### 4.4. Affinity Purification of Biotinylated Proteins

After washing with PBS three times, the cells were lysated with 1 mL of lysis buffer containing 50 mM Tris-HCl, pH7.5, 8M urea, 1 mM DTT, and protein inhibitor cocktail (Merck, Darmstadt, Germany). Lysate was collected into tubes and Tween20 was added at a final concentration of 1%. Following sonication and centrifugation for 5 min at 4 °C and 1000 rpm, 300 µL of the supernatant was transferred to a new tube and incubated with Streptavidin Mag Sepharose (Cytiva, Marlborough, MA, USA) under rotation at room temperature for 30 min. Beads were collected using on a magnetic stand and washed with washing buffer containing TBS, pH7.5, and 2 M urea three times. After washing, beads were stored in washing buffer containing protease inhibitor at −80 °C.

### 4.5. MS Sample Preparation

Following the removal of the washing buffer, beads were suspended in 2 M urea, 50 mM ammonium bicarbonate, and 10 mM DTT and incubated for 30 min at 37 °C with rotation. Iodoacetamide was added at a final concentration of 5% and beads were incubated additionally for 30 min at 37 °C with rotation in the dark. 0.5 µg of Trypsin/LysC Mix (Mass Spec Grade, Promega) was then added and incubated for 3 h at 37 °C with rotation. After diluting the buffer until urea was 1 M, 0.5 µg of Trypsin/LysC Mix was added again and incubated overnight at 37 °C with rotation. The supernatant containing digested peptides was separated from beads using a magnetic stand. The supernatant was desalinated and concentrated through GL-Tip SDB (GL Sciences, Tokyo, Japan). The pellet was suspended with 0.1% TFA and then analyzed in LC-MS/MS.

### 4.6. LC-MS/MS Analysis

The LC-MS/MS measurement was performed with the Q-Exactive hybrid mass spectrometer and the Easy-nLC1000 nanoflow HPLC system (Thermo Fisher Scientific). The trap column used for the nano HPLC was a 2 cm × 75 μm capillary column packed with 3 μm C18-silica particles (Thermo Fisher Scientific), while the separation column was a 12.5 cm × 75 μm capillary column packed with 3 μm C18-silica particles (Nikkyo Technos, Tokyo, Japan). The flow rate of the nano HPLC was 300 nL/min. The separation was conducted using a 10–40% linear acetonitrile gradient for 70 min in the presence of 0.1% formic acid. Each sample was measured three times as technical replicates. The LC-MS/MS data were acquired in the data-dependent acquisition mode controlled using Xcalibur 4.0 (Thermo Fisher Scientific). The settings of the data-dependent acquisition were as follows: the resolutions were 70,000 for the full MS scan and 17,500 for the MS2 scan; the AGC targets were 3.0E6 for the full MS scan and 5.0E5 for the MS2 scan; the maximum IT was 60 msec for both the full MS and MS2 scans; the scan range was 310–1500 *m*/*z* for the full MS scan, with the top 10 signals being selected for the MS2 scan per one full MS scan; and the dynamic exclusion was 15 s.

### 4.7. Data Analysis

Data analysis was performed with the Proteome Discoverer 2.4 software (Thermo Fishier Scientific) bundled with the Sequest HT search engine. The MS/MS spectra were searched against all human ORF sequences obtained from the UniProt database (https://www.uniprot.org/, the accession ID was UP000005640 (containing 20,364 reviewed proteins and their isoforms), accessed on 22 November 2019). The ratio calculation and the hypothesis test were conducted with the “Summed Abundance Based” and “Individual proteins” modes, respectively. Detailed settings of the search with Proteome Discoverer 2.4 are listed in Supplementary Table S1. Only the proteins with a high FDR confidence (<0.01 via Percolator algorithm), annotated as “IsMasterProtein”, and a ≥2 number of peptides were defined as identified proteins and used for the quantitative analysis. The proteins annotated as contaminants were removed.

### 4.8. Flow Cytometry Analyses

HEK293T cells were grown to confluence, resuspended, and seeded onto 35 mm plates at 25% confluency. The cells were cultured overnight and then transfected with the genes in pCI vector. GFP expression vector was also transfected per dish as a control for transfection efficiency. At 24 h post-transfection, the cells were dissociated in Cellstripper^®^ (CORNING, Corning, NY, USA) and transferred to a tube for incubation with the anti-rhodopsin 4D2 antibody (Merck) and APC-conjugated donkey anti-mouse IgG (BioLegend, Sandiego, CA, USA). To stain dead cells, 7-Amino-actinomycin D (Merck) was added. The cells were analyzed using CytoFLEX (Beckman Coulter, Brea, CA, USA) with gating allowing for GFP positive, single, spherical, viable cells; the measured PE intensities were analyzed and visualized using Flowjo.

### 4.9. Split Luciferase Reconstruction Assay

We used Nano-Glo^®^ Live Cell Assay System (Promega) for the split luciferase reconstruction assay. HEK293T cells were plated on poly-d-lysine-coated 96-well plates. Plasmid DNAs were transfected using Viafect. Each protein was sub-cloned into pBiT1.1-C [TK/LgBiT], pBiT2.1-C [TK/SmBiT], pBiT1.1-N [TK/LgBiT], and pBiT2.1-N [TK/SmBiT] vector. Plasmids were transfected into HEK293T cells in different combinations. At 24 h post-transfection, the medium was replaced with 50 µL of D-MEM and we followed the manufacturer’s protocols for measuring luciferase activities. Luminescence was measured using a GloMax^®^-Multi Detection System (Promega).

## Data Availability

Not applicable.

## Supplementary Materials

The supporting information can be downloaded at: https://www.mdpi.com/article/10.3390/ijms24097829/s1.
