# Supplementary material for: Identification and Characterization of Proteins That Are Involved in RTP1S-Dependent Transport of Olfactory Receptors
Source: Int J Mol Sci. 2023 Apr 25;24(9):7829. doi: 10.3390/ijms24097829 (PMC10177996; doi:10.3390/ijms24097829)
Supplement: Supplementary file 1 [file ijms-24-07829-s001.zip › Supplemental Figures.pdf]

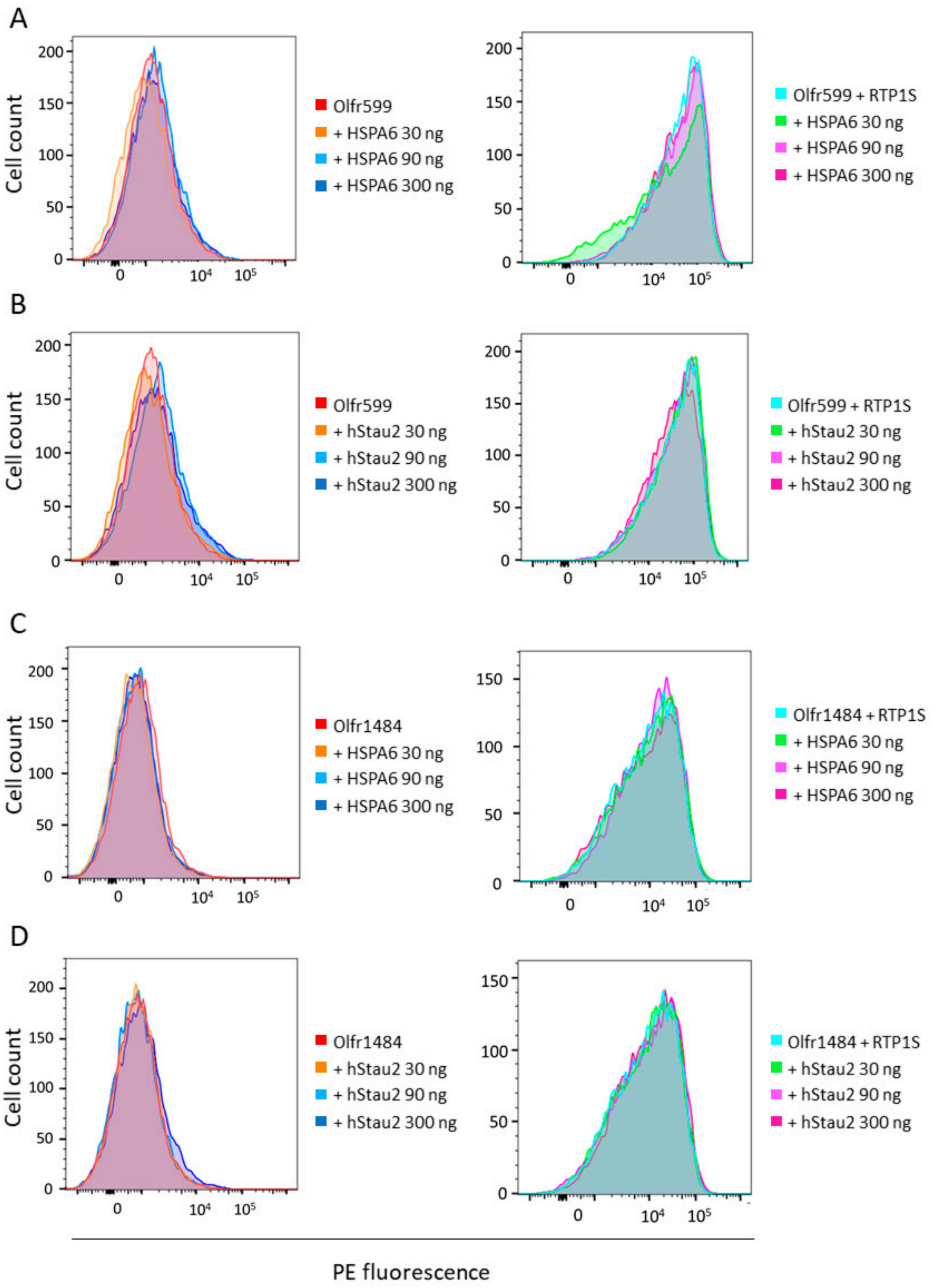

Supplementary Figure 1. Cell surface expression of Olfr599 and Olfr1484 are not increased by co-expression with HSPA6 or hStau2. FACS analysis for cell surface expression of Olfr599 or Olfr1484 co-expressed with a candidate chaperone protein. (A) Olfr599 with HSPA6 (left) or with HSPA6 and RTP1S (right) (B) Olfr599 with hStau2 (left) or with hStau2 and RTP1S (right) (C) Olfr1484 with HSPA6 (left) or with HSPA6 and RTP1S (right) (D) Olfr1484 with hStau2 (left) or with hStau2 and RTP1S (right)

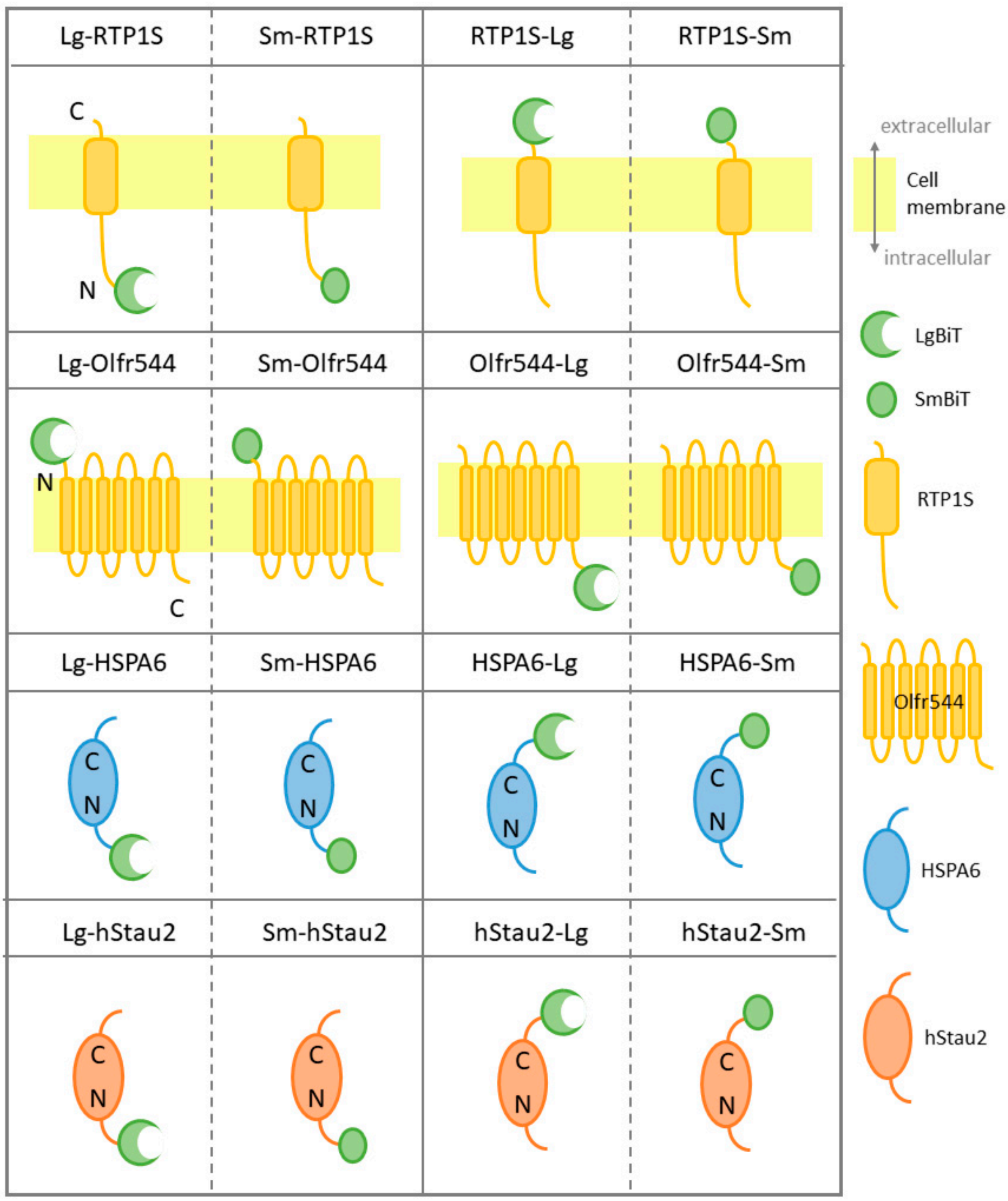

Supplementary Figure S2. Subunits for NanoBiT assay are fused to N-terminus or C-terminus of each protein. Construction of RTP1S, Olfr544, HSPA6 and hStau2 fused with NanoBiT subunit.

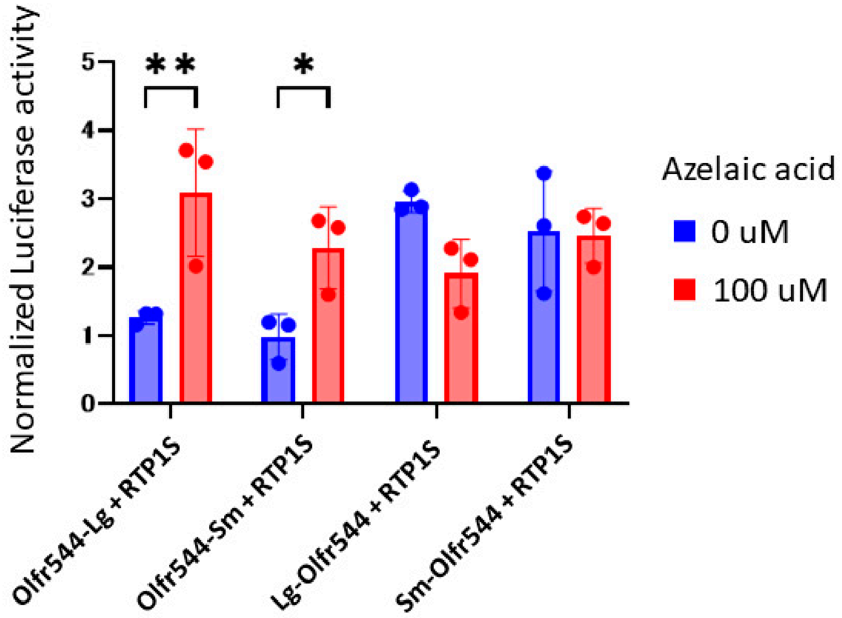

Supplementary Figure. S3 Olfr544-NanoBiT have very slightly low activity of ligand response and NanoBiT-Olfr544 lose that. Dual-Glo luciferase assay were performed to evaluate ligand response activities of Olfr544 fused subunit of NanoBiT. Multiple comparisons were performed using two-way analysis of variance (ANOVA) followed by Sidak test(*$p<0.05$, **$p<0.01$).
