# Supplementary material for: Identification and Characterization of Proteins That Are Involved in RTP1S-Dependent Transport of Olfactory Receptors
Source: Int J Mol Sci. 2023 Apr 25;24(9):7829. doi: 10.3390/ijms24097829 (PMC10177996; doi:10.3390/ijms24097829)
Supplement: Supplementary file 1 [file ijms-24-07829-s001.zip › Supplementary Table S1.pdf]

Supplementary Table S1. Settings of Proteome Discoverer 2.4.

Processing Workflow:

Node	Parameter	Values
Spectrum Files RC	Protein Database	PD_Contaminants_2015_5 (bundled in Proteome Discoverer) All human ORFs (from UniProt: UP UP000005640, downloaded on 191122)
	Enzyme Name	Trypsin (full)
	Dynamic Modification	None
	Static Peptide N-Terminus	None
	Static Modifications	None
Spectrum Selector	1. General Settings	
	Precursor Selection	Use MS1 Precursor
	Provide Profile Spectra	Automatic
	2. Spectrum Properties Filter	
	Lower RT Limit	0
	Upper RT Limit	0
	First Scan	0
	Last Scan	0
	Lowest Charge State	0
	Highest Charge State	0
	Min. Precursor Mass	350 Da
	Max. Precursor Mass	5000 Da
	Total Intensity Threshold	0
	Minimum Peak Count	1
	3. Scan Event Filters	
	MS Order	Is Not MS1
	Min. Collision Energy	0
	Max. Collision Energy	1000
	Scan Type	Is Full
	4. Peak Filters	
	S/N Threshold (FT-only)	1.5
	5. Replacements for Unrecognized Properties	
	Unrecognized Charge Replacements	Automatic
	Unrecognized Mass Analyzer Replacements	ITMS
	Unrecognized MS Order Replacements	MS2
	Unrecognized Activation Type Replacements	CID
	Unrecognized Polarity Replacements	+
	Unrecognized MS Resolution@200 Replacements	60000
	Unrecognized MSn Resolution@200 Replacements	30000
Sequest HT	1. Input Data	
	Protein Database	PD_Contaminants_2015_5 (bundled in Proteome Discoverer) All human ORFs (from UniProt: UP UP000005640, downloaded on 191122)
	Enzyme	Trypsin (full)
	Max. Missed Cleavage Sites	2
	Min. Peptide Length	6
	Max. Peptide Length	100

	2. Tolerances	
	Precursor Mass Tolerance	10 ppm
	Fragment Mass Tolerance	0.02 Da
	Use Average Precursor Mass	False
	Use Average Fragment Mass	False
	3. Spectrum Matching	
	Use Neutral Loss a Ions	True
	Use Neutral Loss b Ions	True
	Use Neutral Loss y Ions	True
	Use Flanking Ions	True
	Weight of a Ions	0
	Weight of b Ions	1
	Weight of c Ions	0
	Weight of x Ions	0
	Weight of y Ions	1
	Weight of z Ions	0
	4. Dynamic Modifications	
	Max. Equal Modifications Per Peptide	3
	Dynamic Modification	Oxidation / +15.995 Da (M)<br>Deamidated / +0.984 Da (N, Q)
	5. Dynamic Modifications (peptide terminus)	
	(None)	
	6. Dynamic Modifications (protein terminus)	
	1. N-Terminal Modification	Acetyl / +42.011 Da (N-Terminus)
	7. Static Modifications	
	1. Static Modification	Carbamidomethyl / +57.021 Da (C)
Percolator	1. Target/Decoy Strategy	
	Target/Decoy Selection	Concatenated
	Validation based on	q-Value
	2. Input Data	
	Maximum Delta Cn	0.05
	Maximum Rank	0
	3. FDR Targets	
	Target FDR (Strict)	0.01
	Target FDR (Relaxed)	0.05
Minora Feature Detector	(No parameters)	

Consensus Workflow:

Node	Parameter	Values
MSF Files	1. Storage Settings	
	Spectra to Store	Identified or Quantified
	Feature Traces to Store	All
	2. Merging of Identified Peptide and Proteins	
	Merge Mode	Do Not Merge
	3. FASTA Title Line Display	
	Reported FASTA Title Lines	Best match
	4. PSM Filters	
	Maximum Delta Mass	0 ppm

PSM Grouper	1. Peptide Group Modifications	
	Site Probability Threshold	75
Peptide Validator	1. General Validation Settings	
	Validation Mode	Automatic (Control peptide level error rate if possible)
	Target FDR (Strict) for PSMs	0.01
	Target FDR (Relaxed) for PSMs	0.05
	Target FDR (Strict) for Peptides	0.01
	Target FDR (Relaxed) for Peptides	0.05
	2. Specific Validation Settings	
	Target/Decoy Selection for PSM Level FDR Calculation Based on Score	Automatic
	Reset Confidences for Nodes without Decoy Search (Fixed score thresholds)	False
Peptide and Protein Filter	1. Peptide Filters	
	Peptide Confidence At Least	High
	Keep Lower Confident PSMs	True
	Minimum Peptide Length	6
	Remove Peptides Without Protein Reference	False
	2. Protein Filters	
	Minimum Number of Peptide Sequences	1
	Count Only Rank 1 Peptides	False
	Count Peptides Only for Top Scored Protein	False
Protein Scorer	(No parameters)	
Protein Grouping	1. Protein Grouping	
	Apply strict parsimony principle	True
Peptide in Protein Annotation	1. Flanking Residues	
	Annotate Flanking Residues of the Peptide	False
	2. Modifications in Peptide	
	Protein Modifications Reported	Only for Master Proteins
	3. Modifications in Protein	
	Modification Sites Reported	All And Specific
	Minimum PSM Confidence	High
	Report Only PTMs	True
	4. Positions in Protein	
	Protein Positions for Peptides	Only for Master Proteins
Protein FDR Validator	1. Confidence Thresholds	
	Target FDR (Strict)	0.01
	Target FDR (Relaxed)	0.05
Protein Marker	1. Contaminant Database	
	Protein Database	PD_Contaminants_2015_5
	5. Annotate Species	
	As Species Map	False
	As Species Names	False
	6. Mark Additional Entities	
	Annotation Groups	False
	Pathway Groups	False
	Modification Sites	True
	Peptide Isoform Groups	True
Feature Mapper	1. Chromatographic Alignment	
	Perform RT Alignment	True

	Maximum RT Shift [min]	10
	2. Feature Linking and Mapping	
	Min. S/N Threshold	5
Precursor Ions Quantifier	1. General Quantification Settings	
	Peptides to Use	Unique + Razor
	Consider Protein Groups for Peptide Uniqueness	True
	Use Shared Quan Results	True
	Reject Quan Results with Missing Channels	False
	2. Precursor Quantification	
	Precursor Abundance Based On	Intensity
	Min. # Replicate Features [%]	0
	3. Normalization and Scaling	
	Normalization Mode	Total Peptide Amount
	Scaling Mode	None
	4. Exclude Peptides from Protein Quantification	
	For Normalization	Exclude Modified
	For Protein Roll-Up	Use All Peptides
	For Pairwise Ratios	Exclude Modified
	5. Quan Rollup and Hypothesis Testing	
	Protein Abundance Calculation	Summed Abundances
	N for Top N	3
	Protein Ratio Calculation	Protein Abundance Based
	Maximum Allowed Fold Change	100
	Imputation Mode	None
	Hypothesis Test	ANOVA (Individual Proteins)
	6. Quan Ratio Distributions	
	1st Fold Change Threshold	2
	2nd Fold Change Threshold	4
	3rd Fold Change Threshold	6
	4th Fold Change Threshold	8
	5th Fold Change Threshold	10
